# Daytime food restriction alters liver glycogen, triacylglycerols, and cell size. A histochemical, morphometric, and ultrastructural study

**DOI:** 10.1186/1476-5926-9-5

**Published:** 2010-02-23

**Authors:** Mauricio Díaz-Muñoz, Olivia Vázquez-Martínez, Adrián Báez-Ruiz, Gema Martínez-Cabrera, María V Soto-Abraham, María C Ávila-Casado, Jorge Larriva-Sahd

**Affiliations:** 1 Instituto de Neurobiología, Campus UNAM-UAQ, Juriquilla, Querétaro, 76001 QRO, México; 2 Instituto Nacional de Cardiología, Juan Badiano #1, Ciudad de México, 14080, DF, México

## Abstract

**Background:**

Temporal restriction of food availability entrains circadian behavioral and physiological rhythms in mammals by resetting peripheral oscillators. This entrainment underlies the activity of a timing system, different from the suprachiasmatic nuclei (SCN), known as the food entrainable oscillator (FEO). So far, the precise anatomical location of the FEO is unknown. The expression of this oscillator is associated with an enhanced arousal prior to the food presentation that is called food anticipatory activity (FAA). We have focused on the study of the role played by the liver as a probable component of the FEO. The aim of this work was to identify metabolic and structural adaptations in the liver during the expression of the FEO, as revealed by histochemical assessment of hepatic glycogen and triacylglycerol contents, morphometry, and ultrastructure in rats under restricted feeding schedules (RFS).

**Results:**

RFS promoted a decrease in the liver/body weight ratio prior to food access, a reduction of hepatic water content, an increase in cross-sectional area of the hepatocytes, a moderate reduction in glycogen content, and a striking decrease in triacylglyceride levels. Although these adaptation effects were also observed when the animal displayed FAA, they were reversed upon feeding. Mitochondria observed by electron microscopy showed a notorious opacity in the hepatocytes from rats during FAA (11:00 h). Twenty four hour fasting rats did not show any of the modifications observed in the animals expressing the FEO.

**Conclusions:**

Our results demonstrate that FEO expression is associated with modified liver handling of glycogen and triacylglycerides accompanied by morphometric and ultrastructural adaptations in the hepatocytes. Because the cellular changes detected in the liver cannot be attributed to a simple alternation between feeding and fasting conditions, they also strengthen the notion that RFS promotes a rheostatic adjustment in liver physiology during FEO expression.

## Background

From an evolutionary perspective, circadian systems have conferred a survival advantage by optimizing behavioral and physiological adaptations to periodic events that occur approximately each 24 h. An ultimate goal of this adaptation is to enhance the reproductive success and life span by allowing more effective access to nutritional resources [1,2]. The vertebrate circadian system results from the coordinated action of a light-entrained master pacemaker located in the suprachiasmatic nucleus (SCN) of the hypothalamus, and a set of subordinated clocks in peripheral organs [3]. The 24-h programs of the central and peripheral oscillators are based on similar, but not identical, molecular transcription-translation feedback loops [4]. The normal timing between the principal and the peripheral clocks can be disrupted when activity, sleep, or feeding patterns are altered [5]. An example of this situation happens when feeding is restricted to short periods of time, particularly in experimental protocols in which food is offered during the daytime to nocturnal rodents. In this condition, the peripheral clocks become independent of SCN rhythmicity, and the circadian system is no longer entrained by light but primarily by the effects of the scheduling of meal-feeding [6,7]. Central to this adaptation is the expression of a food-entrainable oscillator (FEO) that controls, next to the SCN, the 24-h rhythms of behavioral, physiological, and metabolic activities [8].

The FEO is expressed when animals have access to food on restricted schedules (2 to 4 h of mealtime per day over a period of 2 or 3 weeks). The restricted feeding schedule (RFS) increases locomotive activity and arousal during the hours immediately before food access, generating a condition known as food anticipatory activity (FAA) [9]. FAA is characterized by a variety of physiological and behavioral changes in the organism such as: increases in wheel running activity, water consumption, and body temperature, as well as a peak of serum corticosterone [9-11]. So far, the anatomical location of the FEO is unknown, but the physiology of this oscillator is thought to involve the bidirectional communication between specific, energy-sensitive brain areas and nutrient-handling, peripheral organs, especially the liver [8,9,11].

The liver is primarily composed of parenchymal cells or hepatocytes (80% by volume) and four types of non-parenchymal cells: endothelial, Kupffer, Ito, and pit cells. Hepatic tissue is highly specialized and functions as a major effector organ, acting as: 1) principal center of nutrient metabolism, 2) major component of the organism defensive response, 3) control station of the endocrine system, and 4) blood reservoir [12]. The hepatic gland performs a strategic role in the digestive process by receiving the nutrients from the diet and orchestrating their transformation into useful biomolecules to be delivered to other organs and tissues. Hence, the liver is fundamental in the metabolism of carbohydrates, lipids, and all other biomolecules. Hypothalamic and midbrain nuclei are connected via vagal and splanchnic nerves to the liver, allowing the hepatic organ to participate in the control of food intake by sensing and regulating the energy status of the body [13].

FEO expression promotes dramatic changes in the physiology and metabolic performance of the liver [11,14,15]: During the FAA (before food access), there is a prevalence of oxidized cytoplasmic and mitochondrial redox states, an increase in adenine nucleotides levels, an enhanced mitochondrial capacity to generate ATP, and a hypothyroidal-like condition that is not systemic but exclusively hepatic. In contrast, after feeding the hepatic redox state becomes reduced in both cytoplasmic and mitochondrial compartments, the levels of ATP decline, and the level of T_3 _within the liver increases. However, not all the adaptations in the liver during RFS occur before and after food intake. A constant reduction in pro-oxidant reactions (conjugated dienes and lipid peroxides) in most hepatocyte subcellular fractions and a persistent increase in the mitochondrial membrane potential (ΔΨ) are observed along FEO expression [14,16]. In addition, the liver is the organ that displays the fastest shift in the phase of clock-control genes and molecular outputs in response to food access being restricted to daytime in nocturnal rodents [17].

The aim of the present report was to gain further understanding on the structural and histochemical adaptations underlying glycogen and triacylglycerols metabolism in the liver during the FEO expression. Hence, we evaluated these parameters in rats under RFS at three time points and under two feeding conditions: 1) before, 2) during, and 3) after the FAA. Experimental results were also compared with a control group subjected to a simple 24-h period of fasting. We found that during the FAA: 1) A partial reduction of hepatic glycogen and almost a complete disappearance of triacylglycerols in comparison to the 24-h fasted rats; 2) The water content was decreased, but at the same time the cross-sectional area of the hepatocytes augmented; 3) The hepatocyte cytoplasm displayed rounded mitochondria bearing very electron-dense matrices and a hypertrophy of the smooth endoplasmic reticulum.

## Results

### Somatometry

Table 1 shows the values of body weight reached by the control and experimental animals. After 3 weeks, control groups fed *ad libitum *reached corporal weights between 320 and 340 g, which represented an increase of ≈ 120% over their weight at the beginning of the experiment (≈ 150 g). No significant differences were detected among the three times tested (08:00, 11:00, and 14:00 h). The other control group, the 24-h fasting rats, showed a moderate diminution in body weight of 10%. In contrast, rats under RFS showed significantly lower body weights, 180-195 g before feeding (08:00 and 11:00 h) and 242-251 g after feeding (14:00 h). Considering the initial weight of ≈ 150 g, the values corresponded to an increase in corporal weight of ≈ 25% before feeding and ≈ 64% after feeding. These data indicate that the rats under RFS show a daily oscillation of approximately one third of their weight due to the marked hyperphagia displayed and the water drunk in the 2-h period when they have access to food. The results of body weights clearly show that the animals under RFS were smaller than control rats fed *ad libitum*, but at the same time, they also indicate that our experimental protocol did allow a slight growth in the RFS rats.

**Table 1:** Change of body weight (BW) of rats after 3 weeks under restricted feeding schedules.

Treatment	Initial BW (g)	Final BW (g)	Δ BW (%)
***Food ad libitum***			
08:00 h	151 ± 3	320 ± 21	169 (112%)
11:00 h	150 ± 2	329 ± 26	179 (119%)
14:00 h	153 ± 2	337 ± 31	184 (120%)
***Food restricted schedule***			
08:00 h	150 ± 2	182 ± 17*	32 (21%)*
11:00 h	151 ± 3	192 ± 20*	41 (27%)*
14:00 h	149 ± 1	246 ± 23* ^+^	97 (65%)* ^+^
***24 h Fasting***			
11:00 h	321 ± 4	298 ± 3	-23 (-7%)

Values are means ± SE for 6 independent observations. Male Wistar rats were under food restriction for three weeks. Food access from 12:00 to 14:00 h. Control groups included rats fed *ad libitum *and rats fasted for 24 h. Results are expressed as mean ± SEM of 6 independent determinations. Significant difference between RFS and *ad-libitum *groups (*), within the same experimental group (+), and different from 24-h fasting group (x). Differences derived from to Tukey's post hoc test (α = 0.05).

Table 2 shows the changes in the liver weight and the ratio liver/body weight reached by the control and experimental animals. The liver weight showed no significant variation among the 3 control groups of rats fed *ad libitum*, and the value of the ratio liver/body weight (4.2 ± 0.1) was in the range reported previously [18]. Fasting for 24 h decreased the liver weight by ≈ 30%, making the ratio liver/body weight (3.2 ± 0.1) smaller than those obtained in rats fed *ad libitum*. This effect had been already reported [19]. The liver weights in the RFS groups were significantly lower at the 3 times studied: Before feeding (08:00 and 11:00 h) the value corresponded to a decrease of ≈ 55% in comparison with the *ad-libitum *fed group; after feeding (14:00 h) the reduction in the liver weight was ≈ 41%. At the 3 times studied, and independently of the food intake, the ratio liver/body weight in the rats under RFS was lower than in the groups fed *ad libitum*, and similar to the 24-h fasted group (3.1 ± 0.1). These data imply that RFS promotes a sharper drop in liver weight than in body weight, similar to the effect on 24-h fasted rats. Interestingly, after 2 h feeding, rats under RFS showed an increase of ≈ 30% in the weight of liver and body (comparing groups at 11:00 and 14:00 h).

**Table 2:** Liver weigth (LW) and ratio LW/body weight of rats under food restricted schedules.

Treatment	LW (g)	LW/BW × 100
***Food ad libitum***		

08:00 h	13.5 ± 0.8	4.2 ± 0.2

11:00 h	13.8 ± 0.6^×^	4.1 ± 0.3^×^

14:00 h	14.7 ± 0.9	4.3 ± 0.1

***Food restricted schedule***		

08:00 h	6.5 ± 0.2*	3.6 ± 0.3*

11:00 h	6.1 ± 0.3*	3.2 ± 0.2*

14:00 h	8.2 ± 0.4*	3.3 ± 0.2*

***24 h Fasting***		

11:00 h	9.7 ± 0.3	3.2 ± 0.3

Values are means ± SE for 6 independent observations. Male Wistar rats were under food restriction for three weeks. Food access from 12:00 to 14:00 h. Control groups included rats fed *ad-libitum *and rats fasted for 24 h. Results are expressed as mean ± SEM of 6 independent determinations. Significant difference between RFS and *ad-libitum *groups (*), and different from 24-h fasting group (x). Differences derived from Tukey's post hoc test (α = 0.05). BW = body weight.

### Liver water content (LWC)

**Figure 1:**
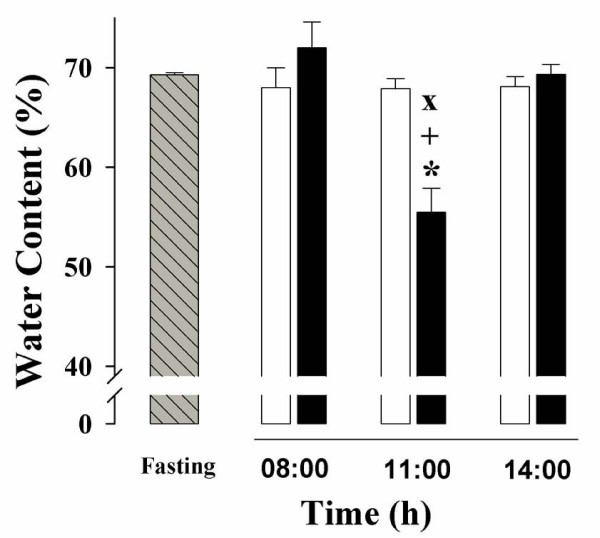
**Water content in the liver of rats exposed to a restricted feeding schedule for 3 weeks (food intake from 12:00 to 14:00 h)**. Experimental group, black box; *ad-libitum *fed control group, white box; 24-h fasting control group, hatched and gray box. Data were collected before (08:00 h), during (11:00 h), and after food anticipatory activity (14:00 h). Control group with 24-h fasting was processed at 11:00 h. Results are expressed as mean ± SEM of 6 independent determinations. Significant difference between food-restricted and *ad-libitum *fed groups [*], within the same experimental group at different times [+], and different from 24-h fasting group [×]. Differences derived from Tukey's post hoc test (α = 0.05).

The percentage of water in hepatic tissue varies according to circadian patterns and as a function of food availability [20,21]. LWC was quantified by weighting the dried out tissue (Figure 1). The values obtained for the control and most of the experimental groups varied in a narrow range (68-72%), which matches the LWC reported previously [21]. The only group that showed a significant change was the RFS rats prior to food presentation (11:00 h), and hence, displaying the FAA. The livers of these rats had a water content of only 56%, a 20% decrease compared to the *ad-libitum *fed control, the 24-h fasted rats, and the other two groups of rats under RFS (08:00 and 14:00 h). As reported previously for other parameters, this result suggests that the liver response during fasting associated with RFS is qualitatively different from that during a single fasting period of 24 h.

### Hepatocyte morphometry

**Figure 2:**
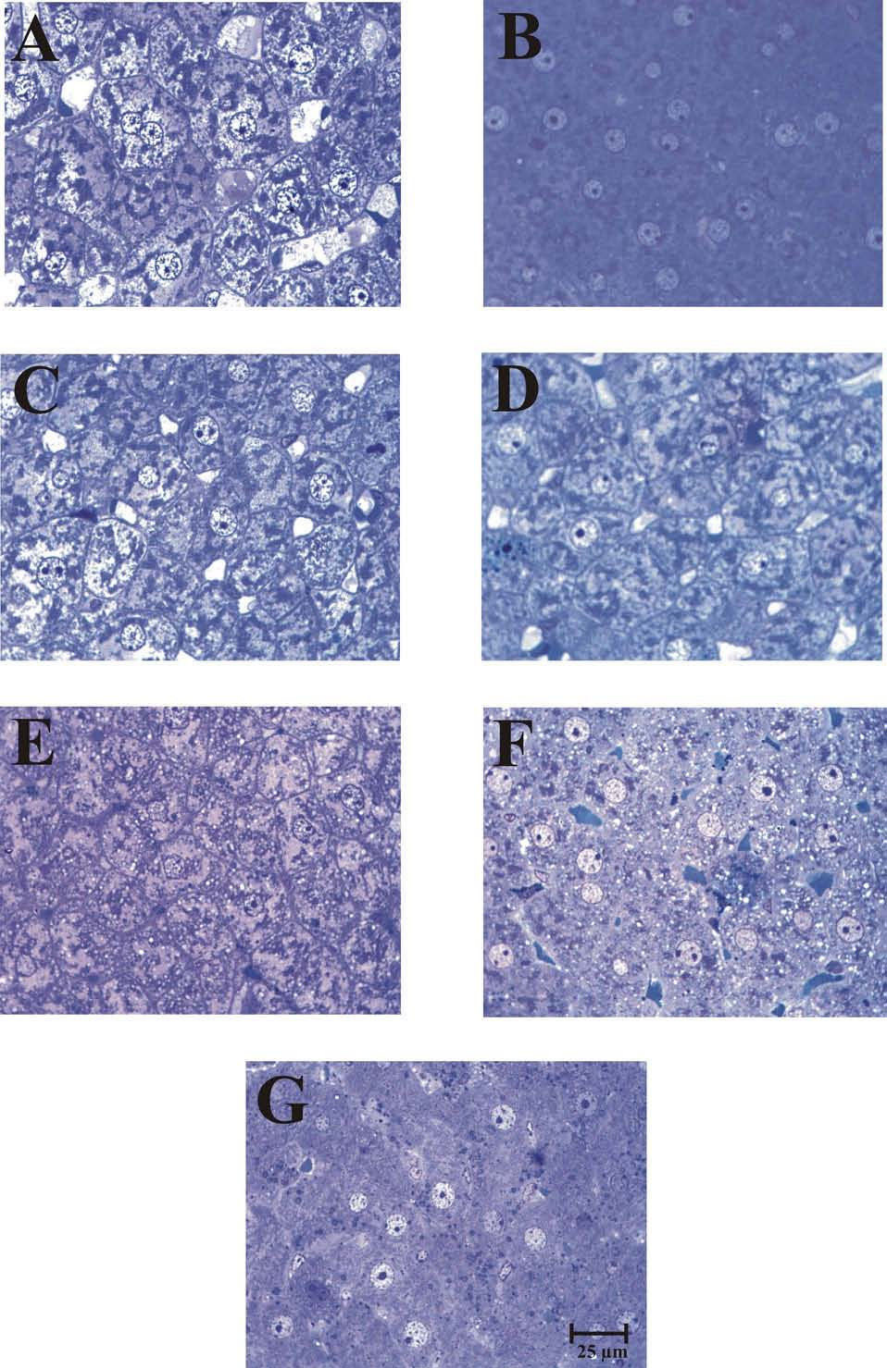
**Toluidine blue-stained histological sections of livers of rats exposed to a restricted feeding schedule for 3 weeks (food intake from 12:00 to 14:00 h)**. Tissue samples from food-restricted and *ad-libitum *fed rats were collected before (08:00 h), during (11:00 h), and after food anticipatory activity (14:00 h). The control group with 24-h fasting was processed at 11:00 h. Panels A, C, and E, control *ad-libitum *fed groups; panels B, D, and F, food-restricted groups; panel G, 24-h fasted group. Images in panels A and B were taken at 08:00 h, in panels C, D and G at 11:00 h, and E and F at 14:00 h.

**Figure 3:**
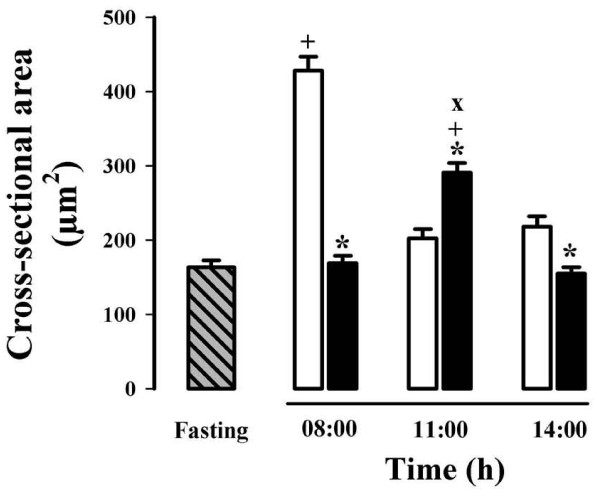
**Quantification of the hepatocytes' cross-sectional area of rats exposed to a restricted feeding schedule for 3 weeks (food intake from 12:00 to 14:00 h)**. Data are derived from evaluation of the hepatocyte morphology (Figure 2). RFS group, black box; ad-libitum-fed control group, white box; 24-h-fasting control group, hatched and gray box. Results are expressed as mean ± SEM of 6 independent determinations. Significant difference between food restricted and *ad-libitum *fed groups [*], within the same experimental group [+], and different from 24-h fasting group [×]. Differences derived from Tukey's post hoc test (α = 0.05).

It has been shown that dietary state influences the hepatocyte dimensions [22]. Histological preparation and morphometric examination of hepatic tissue demonstrated striking changes in the cross-sectional area (as a proxy of cell 3D size) of liver cells between control rats fed *ad libitum *and rats under RFS (Figures 2 and 3). Only hepatocytes displaying a distinct nucleus and at least one nucleolus were included in the morphometric analysis. Rats fed *ad libitum *showed a significant enhancement in hepatocyte size at 08:00 h (at the end of the feeding period): the increases in surface area was ≈ 100% in comparison to the groups fed *ad libitum *at 11:00 and 14:00 h (Figure 2, panels A, C, and E). The group with 24-h of fasting showed no variation in the size of their liver cells compared to the *ad-libitum *fed counterpart (at 11:00 h) (Figure 2, panels C and G). Food restriction also promoted obvious modifications in hepatocyte morphometry: Coincident with the FAA, at 11:00 h, hepatocytes cross-sectional area increased ≈ 53% in relation to the RFS groups before (08:00 h) and after the FAA (14:00 h) (Figure 2, panels B, D, and F). The increased size of the hepatocyte during FAA was also statistically significant when compared to the 24-h fasted rats at 11:00 h (Figure 2, panels D and G). In contrast to the group fed *ad libitum *that showed larger hepatocytes after mealtime (at 08:00 h), the liver cells of the rats expressing the FEO were larger before food intake (at 11:00 h).

### Liver glycogen

**Figure 4:**
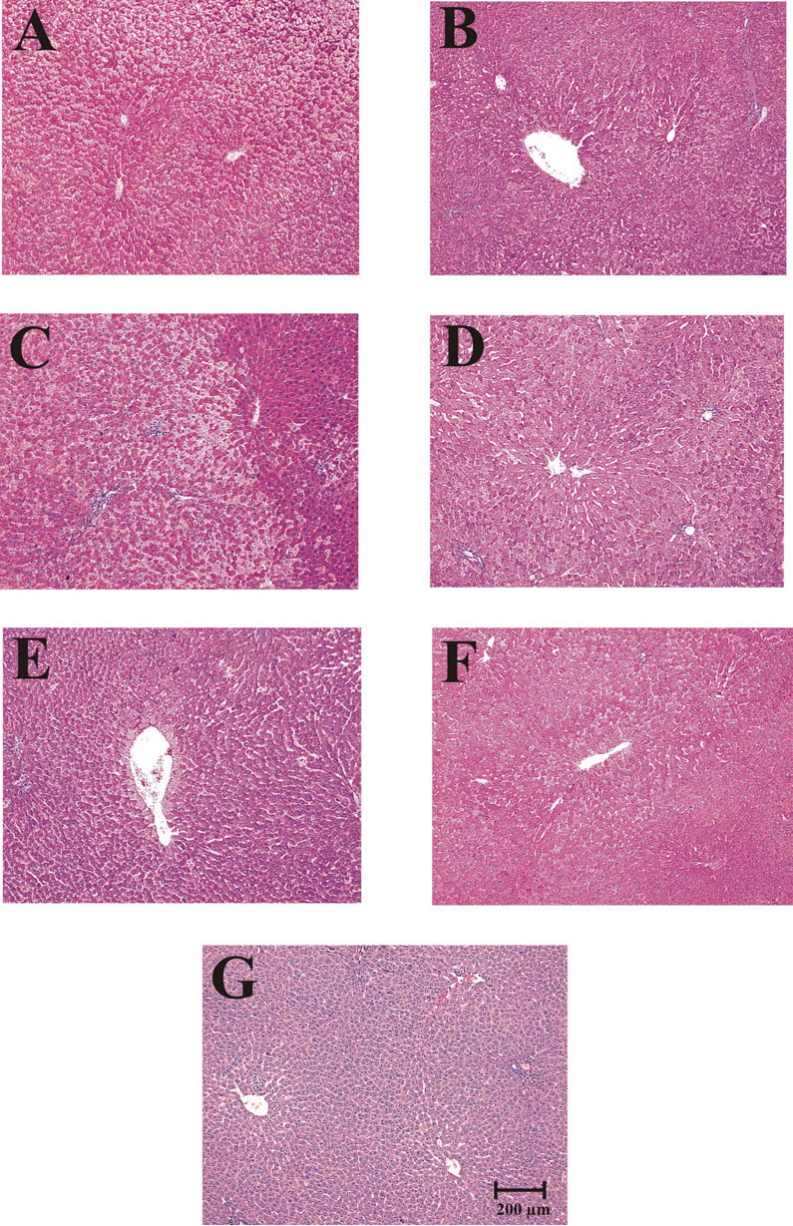
**Periodic-acid Schiff (PAS) stained histological sections of livers of rats exposed to a restricted feeding schedule for 3 weeks (food intake from 12:00 to 14:00 h)**. Pink color indicates the presence of hepatic glycogen. Tissue samples from food-restricted and *ad-libitum *fed rats were collected before (08:00 h), during (11:00 h), and after food anticipatory activity (14:00 h). The control group with 24-h fasting was processed at 11:00 h. Panels A, C, and E, control ad-libitum fed groups; panels B, D, and F, food-restricted groups; panel G, 24-h fasted group. Images in panels A and B were taken at 08:00 h, in panels C, D and G at 11:00 h, and E and F at 14:00 h.

**Figure 5:**
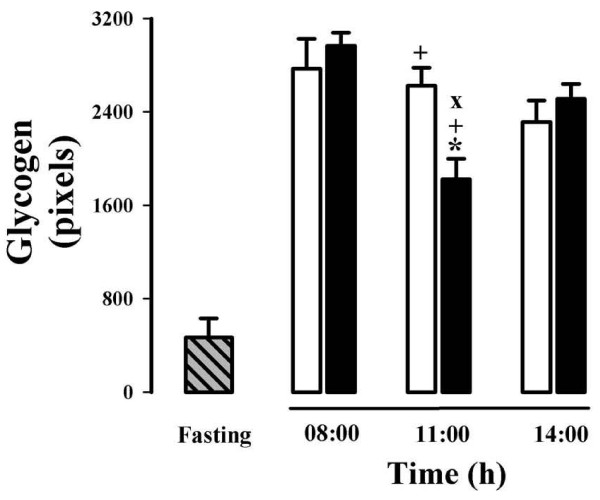
**Quantification of the hepatocytes' glycogen content of rats exposed to a restricted feeding schedule for 3 weeks (food intake from 12:00 to 14:00 h)**. Data are derived from evaluation of the liver PAS staining from Figure 4. RFS group, black box; ad-libitum-fed control group, white box; 24-h-fasting control group, hatched and gray box. Results are expressed as mean ± SEM of 6 independent determinations. Significant difference between food restricted and *ad-libitum *fed groups [*], within the same experimental group [+], and different from 24-h fasting group [×]. Differences derived from Tukey's post hoc test (α = 0.05).

The presence of glycogen in the cytoplasm of hepatocytes was detected and quantified using the periodic acid-Schiff (PAS) staining (Figures 4 and 5). Glycogen staining intensity remained mostly constant in the groups of rats fed *ad libitum *(Figure 4, panels A, C, and E, and Figure 5), with a slight tendency for glycogen levels to decline in the rats at 14:00 h (Figure 5). The group with 24-h fasting showed a dramatic reduction (≈ 82%) in the glycogen content (Figure 4, panel G, and Figure 5). Rats under RFS showed a significant but smaller decrease in liver glycogen (≈ 30%) during the FAA (at 11:00 h). Indeed, the reduction in glycogen in the rats expressing the FEO was less than that shown by the 24-h fasted rats, even though both groups had a similar period of fasting (Figure 4, panels D and G, and Figure 5). After food ingestion (at 14:00 h), hepatic glycogen in RFS rats reverted to normal levels.

### Liver triacylglycerols

**Figure 6:**
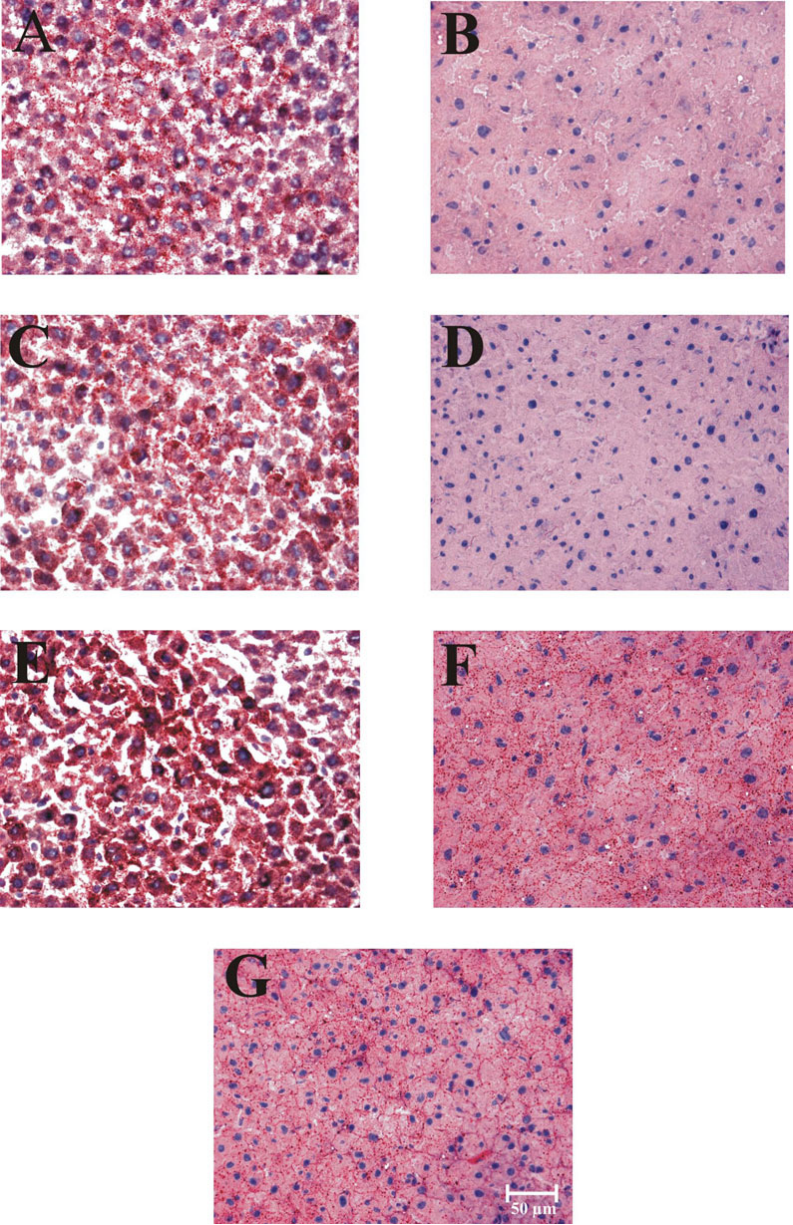
**Oil red O (ORO)-stained histological sections of livers of rats exposed to a restricted feeding schedule for 3 weeks (food intake from 12:00 to 14:00 h)**. Intense red color indicates the presence of neutral lipids, mainly triacylglycerols. Tissue samples from food restricted and *ad-libitum *fed rats were collected before (08:00 h), during (11:00 h), and after food anticipatory activity (14:00 h). Control group with 24-h fasting was processed at 11:00 h. Panels A, C, and E, control *ad-libitum *fed groups; panels B, D, and F, food-restricted groups; panel G, 24-h fasted group. Images in panels A and B were taken at 08:00 h, in panels C, D and G at 11:00 h, and E and F at 14:00 h.

**Figure 7:**
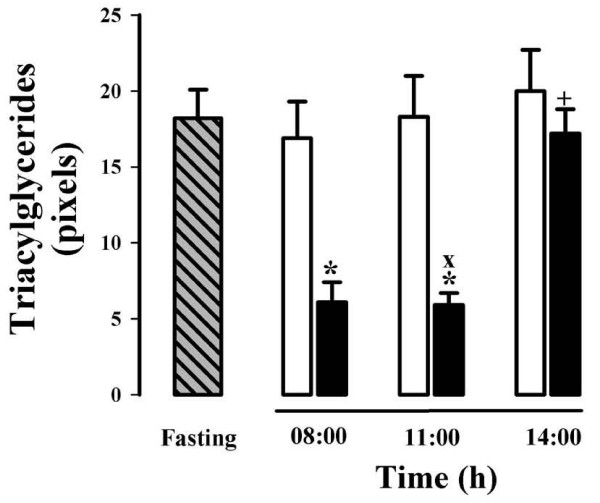
**Quantification of the hepatocytes' triacylglycerols content of rats exposed to a restricted feeding schedule for 3 weeks (food intake from 12:00 to 14:00 h)**. Data are derived from evaluation of the liver oil red O staining from Figure 6. RFS group, black box; ad-libitum-fed control group, white box; 24-h-fasting control group, hatched and gray box. Results are expressed as mean ± SEM of 6 independent determinations. Significant difference between food restricted and *ad-libitum *fed groups [*], within the same experimental group [+], and different from 24-h fasting group [×]. Differences derived from Tukey's post hoc test (α = 0.05).

Neutral hepatic lipids, mainly triacylglycerols, were detected and quantified in frozen liver sections using the oil red O (ORO) stain (Figures 6 and 7). Similar to the results of hepatic glycogen, triacylglycerols did not change in the livers of the groups fed *ad libitum *(Figure 6, panels A, C, and E, and Figure 7). Only an increasing trend was observed in the staining signal in the group at 14:00 h (Figure 7). In contrast to the glycogen results, 24 h of fasting did not modify the hepatic triacylglycerol levels (Figure 6, panel G). Remarkably, the rats under RFS presented much lower triacylglycerol values before food access (08:00 and 11:00 h, Figure 6, panels B and D, and Figure 7). At both times the diminution was very significant (≈ 70%) in relation to their *ad-libitum *fed controls and to the rats with 24-h fasting. After feeding (at 14:00 h), the triacylglycerol content in the food-restricted rats returned to the control levels (Figure 6, panel F and Figure 7). This result supports the notion that an altered processing of lipids in liver, adipose tissue, and transport in blood (high levels of circulating free fatty acid and ketone bodies during the FAA) is established during the FEO expression [10].

### Hepatocyte ultrastructure

**Figure 8:**
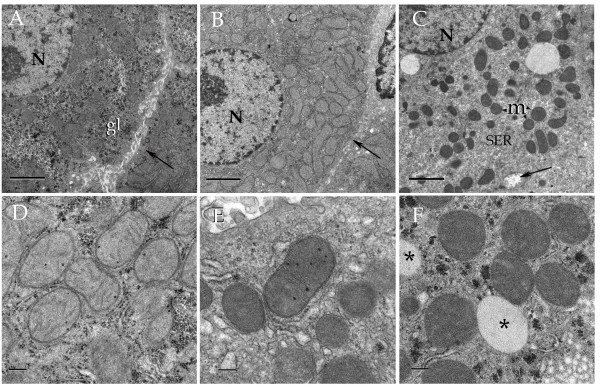
**Electron micrographs illustrating liver cells from control (A and D) fasten (B and D) and fed restricted (C and E) rats**. Notice that hepatocytes from the fed restricted animal (F) exhibit electron-dense mitochondria (m) surrounded by abundant smooth endoplasmic reticulum (SER). N = cell nucleus, gl = glycogen, asterisks = lipid droplets, arrows = bile canaliculi. Lead-uranium staining. Scale bars = 2 μm in A-C; 0.2 μm in D-E. Representative images of 6 independent experimental observations.

Electron microscopic analysis was performed in samples from rats sacrificed at 11:00 h, including: 1) control rats fed *ad libitum*, 2) rats under RSF and displaying the FAA, and 3) control rats with a simple 24-h period of fasting. Figure 8 shows ultrastructural features of hepatocytes from rats subjected to these treatments at low (panels A, B, and C) and high (panels D, E, and F) magnification. Hepatocytes from rats fed *ad libitum *contained numerous mitochondria, well-defined endoplasmic reticulum and nucleus, as well as abundant glycogen deposits in the form of electron-dense material (panels A and D). All glycogen aggregates disappeared after 24 h of fasting, with no further alteration in the structure of the other organelles (Panel B and E). In contrast, hepatocytes from rats during the FAA showed remarkable changes, including an increased opacity that made the cristae difficult to distinguish. Some glycogen was also observed in these hepatocytes, supporting the result obtained with the PAS stain (panels C and F).

## Discussion

The liver is the principal organ that processes nutrients and delivers metabolites to peripheral tissues and organs; hence, it plays a key role in regulating the energy balance of vertebrates and thereby is fundamental in the physiological control of the hunger-satiety cycle [23]. Because feeding determines the individual viability, the timing of the underlying internal metabolic and cellular mechanisms to find and ingest food is properly regulated by circadian systems [24]. In consequence, a variety of liver functions related to the handling of nutrients are targets of circadian control [25]. For these reasons, the hepatic involvement has been considered as an important constituent of the FEO [8,11,17]. Indeed, the FEO expression also depends on the nutritional properties and the caloric content of the meal offered during the RFS [26].

Many of the adaptations in the biochemical responses of the liver before and after feeding during the FEO expression are unique, and do not correspond to the characteristics shown in either control group: fed *ad libitum *or 24-h fasting [10,11,14-16]. Taken together, the data strongly suggest that FEO physiology is associated with a new rheostatic equilibrium in the functional and structural properties of the liver that adapt to optimizing the handling of nutrients under the RSF status [11,15,27].

The liver exhibits daily fluctuations in structural and metabolic features, usually associated with the intake and processing of nutrients from the diet. This oscillatory pattern involves daily adjustments in the hepatocyte function to achieve a suitable assimilation of food, and then a correct processing of nutrients [28]. RFS leads to a striking hyperphagia that result in the ingestion of ≈ 30 g of food during the mealtime. By the time the stomach is almost empty, the FAA begins [29]. It has been reported that, following the rhythm of gastric emptying, the weight of the liver shows a clear circadian rhythm with a peak at 08:00 h [20,30]. Although our results did not show differences in the liver weight in the control groups fed *ad libitum *(Table 1), the hepatocytes cross-sectional area was notably bigger at 08:00 h (Figure 2 and Figure 3), suggesting an increase in cell size. Interestingly, the ratio liver weight/body weight was lower at all three times tested in the rats expressing the FEO and similar to the value for the rats fasted 24 h (Table 2), indicating that under RFS, the changes in corporal and liver weights are proportional, before and after feeding. In contrast, in the 24-h fasted group there was a more pronounce reduction in the liver weight, confirming data previously reported [30].

Tongiani et al., have reported a circadian rhythm for the water content in rat hepatocytes with a peak during the night, being the rhythm mainly regulated by the light-dark regimen and not by the time of food access [21]. In our RFS protocol, the only significant variation detected was lower water content during the FAA (at 11:00 h) (Figure 1). At this time, there is intense metabolic activity in the liver characterized by increased mitochondrial respiration, an enhanced ATP synthesis, and a switch from a carbohydrate- to a lipid-based metabolism [10,11,14,31]. We do not know the cellular constituent responsible for the increase in the hepatic dry mass during FAA, but we can rule out glycogen, triacylglycerols and protein content since the first two were present at lower levels during the FAA (Figures 5 and 7), and the letter did not show significant changes [14]. It is noteworthy that at this time (11:00 h), the hepatocyte cross-sectional area was larger in the RFS group (Figure 2 and Figure 3). Hence, during the FAA, and in preparation for receiving and processing the nutrients from the 2-h food consumption, the liver hepatocytes become most likely larger and contain less water.

No circadian rhythmicity has been detected for the hepatic content of glycogen and triacylglycerols, since these two parameters respond exclusively to food intake and the elapsed time in fasting [10,30,31]. RFS groups before food access (08:00 and 11:00 h) showed just a moderate diminution in hepatic glycogen, but a severe reduction in the content of triacylglycerols (Figures 4 and 5). A possible explanation for the smaller decrease in glycogen is the long time required for the stomach to empty (≈ 20-21 h) in this group. As to the lower level of triacylglycerols, experimental evidence shows that in the time preceding food access (11:00 h), the liver is actively metabolizing lipids, as supported by the high level of circulating free fatty acids and ketone bodies, as well as by the expression of lipid-oxidizing peroxysomal and mitochondrial enzymes detected by microarray assays [10,32]. One possibility is that the energy needed for the liver metabolic activity before food access is obtained by consuming the mobilized lipids from the adipose tissue. (In support of this possibility, unpublished results from our laboratory suggest that lipid-mobilizing factors such as PPARα and γ are increased in the liver during the FAA.)

Uhal and Roehrig reported that the dietary state influences the hepatocyte size and volume: 48 h of fasting resulted in a two-fold reduction in hepatocyte size and its protein content, whereas refeeding promoted a 70-80% [22]. Our results reproduced the difference in cross-sectional area between the hepatocytes from *ad-libitum *fed and 24-h fasting rats (Figure 2), but no difference in protein content was detected [14], perhaps because our protocol involved only 24 of fasting. It is noteworthy that the liver cells increased the cross-sectional area during the FAA (11:00 h). This larger size is not linked to a net hepatic biosynthetic activation in the rats displaying FAA, since there is a concurrent drop in the water content of the liver (Figure 1) without changes in protein content [14].

Finally, our electron microscopic observations support and expand the early notion that the hepatocyte structure also fluctuates in circadian and daily rhythms [33].

## Conclusion

We conclude that uncoupling the rat liver circadian activity from the SCN rhythmicity by imposing a feeding time restricted to daylight induces adaptations in the size, ultrastructure, as well as glycogen and triacylglycerols content in hepatocytes. Moreover, the main adaptations caused by the RFS occurred during the FAA, and could be accounted for as a "cellular and metabolic anticipation" by the liver in preparation for processing more efficiently the ingested nutrients. Finally, the unique characteristics of the hepatic response during RFS, which was different from the responses of the *ad-libitum *fed and 24-h control groups, support the notion of a new rheostatic state in the liver during FEO expression.

## Methods

### Animals and housing

Adult male Wistar rats weighing ≈ 150 g at the beginning of the experiment were maintained on a 12:12 h light-dark cycle (lights on at 08:00 h) at constant temperature (22 ± 1°C). The light intensity at the surface of the cages averaged 350 lux. Animals were kept in groups of five in transparent acrylic cages (40 × 50 × 20 cm) with free access to water and food unless stated otherwise. All experimental procedures were approved and conducted according to the institutional guide for care and use of animals under biomedical experimentation (Universidad Nacional Autónoma de México).

### Experimental design

**Figure 9:**
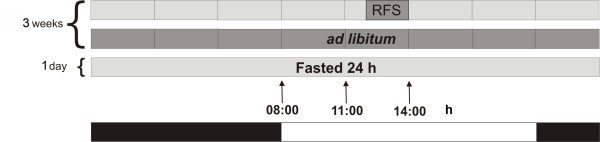
**Time of treatment, feeding conditions, times of sampling and light - darkness cycle used in the experimental protocol**. RFS = restricted feeding schedule.

The experimental procedure reported by Davidson and Stephan [34] was followed with some modifications (Figure 9) [14,15]. Rats were randomly assigned to one of three experimental groups: 1) control rats fed *ad libitum*, 2) rats exposed to a restricted feeding schedule (RFS group) with food presented daily from 12:00 to 14:00 h for three weeks, or 3) control rats with a fast of 24 h. To obtain liver samples, rats from groups 1 (fed *ad-libitum*) and 2 (RFS) were randomly sacrificed at 08:00 h (before FAA), 11:00 h (during FAA), and 14:00 h (after feeding and without FAA). Rats fasted 24 h were killed, and their liver samples removed at 11:00 h. Each experimental group contained 6 rats.

### Liver sampling

Each animal was deeply anesthetized with Anestesal^® ^(sodium pentobarbital) at a dose of 1 ml per 2.5 kg of body weight. In one set of experiments the rats were killed by decapitation, and their livers removed and weighed. A fragment (0.3 - 0.5 g) was weighed, then kept at ≈ 65°C for one week and weighed again; the initial water content was calculated as the difference between the initial and final weights. In a different set of experiments, small sections of each liver were rapidly removed and cut into pieces of about 1 mm^3 ^with sharp razors to be fixed for morphometric measurements and histochemical techniques or processed for electron microscopy.

### Morphometry

Small tissues blocks (≈ 1 mm^3^) for each rat, 6 per group, were immediately fixed in a cold solution of 2.5% glutaraldehyde diluted in 0.15 M cacodylate buffer, pH 7.3. After 60 min, tissues were postfixed for 1 h in 1% osmium tretroxide dissolved in the same buffer. Then, liver fragments were dehydrated in graded acetone dissolved in deionized water and embedded in epoxy resin. One-micron thick semi-thin sections were obtained by a Leica ultramicrotome equipped with glass knives and stained with toluidine blue. Observations were done in a Nikon Eclipse E600 microscope, and images were obtained with a digital camara Photometrics Cool SNAP. Hepatocytes with a single, clear nucleus were selected, and their surfaces were measured with the program IPLab V 3.6 for cross-sectional area determination.

### Histochemical techniques

For glycogen staining, liver fragments (6 rats for each experimental group) were immediately placed and kept 48 h in a fixative (freshly prepared 10% w/v formaldehyde in 0.1 M phosphate buffer, pH 7.2), embedded in paraffin, sectioned at 5-μm thickness, and assessed to detect the content of glycogen within the hepatocytes by the periodic acid-Schiff reaction, with diastase addition for non-specific staining (PAS/D). In this method periodate oxidizes the hydroxyl moieties of glucose residues to aldehydes, which in turn react with the Schiff reagent generating a purple-magenta color. Ten representative fields from at least 4 different liver fragments per rat were analyzed by light microscopy (Olympus BX51; Olympus American, Melville, NY) and captured with a digital video camera (Cool Snap Pro, Media Cybernetics, Silver Spring, MD). Each digital image was photographed with the ×10 objective and formatted at fixed pixel density (8 × 10 inches at 150 dpi) using Adobe Photoshop software (v. 5.5). Each digital image was then analyzed using the MetaMorph Imaging Processing and Analysis software (v. 4.6) for histomorphometric analysis. Glycogen signal was expressed as a percentage of total tissue area. The area of total tissue and the area positively stained for glycogen were calculated in terms of pixels by a co-localization function of the MetaMorph program. Background staining was calculated from slices treated with diastase.

To stain lipids within the hepatocytes, the liver fragments (6 rats for each experimental group) were immediately frozen in solid CO_2_, and the tissue was processed according to the oil red O (ORO) technique. This dye acts not by dissolution but by an adsorption process that gives an intense red stain with fatty acids, cholesterol, triacylglycerols, and unsaturated fats. The quantification of the signal was similar to the one reported in the previous paragraph for glycogen, with the exception that the images were photographed with the ×40 objective.

### Electron microscopy

Liver tissue samples for each rat, 6 per group, were obtained during the laparatomy and cut into about one-millimeter thick blocks, immersed in Karnovsky's fixative (4% paraformaldehyde-2.5% glutaraldehyde in 0.15 M phosphate buffer, pH 7.3) for one hour, washed in the same buffer and stored overnight at 4°C. The next day tissues was postfixed for 1 h in 1% osmium tetraoxide dissolved in the phosphate buffer (vide supra), dehydrated in graded ethyl-alcohols, and embedded in epoxy resin. One-micrometer-thick sections were obtained from the tissue blocks in a Leica ultramicrotome equipped with glass knives. The sections were stained with toluidine blue and coverslipped. From the surface of these trimmed blocks, ultrathin sections ranging from 80 to 90 nm were obtained with a diamond knife and mounted in single-slot grids that had previously been covered with formvar film. The sections were double stained with aqueous solutions of uranium acetate and lead citrate and observed in a JEOL 1010 electron microscope.

### Data analysis

Data were classified by group and time and reported as mean ± SEM. Data from *ad-libitum *and food-restricted groups were compared with a two-way ANOVA for independent measures with a factor for group (2 levels) and a factor for time (6 levels). One-way ANOVA was used to determine significant oscillations in the temporal pattern (6 levels) in each group. All ANOVAs were followed by a Tukey post hoc test with the threshold for significant values set at *p *< 0.05. Values from the fasted rats were compared with those from the group of rats fed *ad libitum *and the rats with restricted feeding sacrificed at 11:00 h, using a one-way ANOVA for independent measures. Statistical analysis was performed with Statisca version 4.5 (StatSoft, 1993).

## Competing interests

The authors declare that they have no competing interests.

## Authors' contributions

MD-M conceived the study, participated in designing the project and drafting the manuscript. OV-M carried out the histological techniques, participated in organizing and analyzing the experimental data, and assembled the figures. AB-R did the initial liver sampling, participated in the histological processing and drafting the manuscript. GM-C participated in the morphometric studies. MVS-A participated in measuring the glycogen and triacylglycerol levels. MCA-C participated in measuring the glycogen and triacylglycerol levels. JL-S participated in designing the project and drafting the manuscript. All authors have read and approved the final article.
